# Diffusion in the corpus callosum predicts persistence of clinical symptoms after mild traumatic brain injury, a multi-scanner study

**DOI:** 10.3389/fnimg.2023.1153115

**Published:** 2023-10-31

**Authors:** Alexander Asturias, Thomas Knoblauch, Alan Rodriguez, Cheryl Vanier, Caroline Le Tohic, Brandon Barrett, Matthew Eisenberg, Rachelle Gibbert, Lennon Zimmerman, Shaunaq Parikh, Anh Nguyen, Sherwin Azad, Leo Germin, Enrico Fazzini, Travis Snyder

**Affiliations:** ^1^Imgen Research Group, Las Vegas, NV, United States; ^2^Touro University Nevada, Henderson, NV, United States; ^3^School of Integrated Health Sciences, University of Nevada Las Vegas, Las Vegas, NV, United States; ^4^Kirk Kerkorian School of Medicine at UNLV, Las Vegas, NV, United States; ^5^Southwest Medical Center, Charleroi, PA, United States; ^6^MountainView Hospital, HCA Healthcare, Las Vegas, NV, United States; ^7^Clinical Neurology Specialists, Las Vegas, NV, United States; ^8^SimonMed Imaging, Scottsdale, AZ, United States

**Keywords:** concussion, post-concussion syndrome (PCS), diffusion tensor imaging (DTI), clinical outcomes, interval-censored

## Abstract

**Background:**

Mild traumatic brain injuries (mTBIs) comprise 80% of all TBI, but conventional MRI techniques are often insensitive to the subtle changes and injuries produced in a concussion. Diffusion tensor imaging (DTI) is one of the most sensitive MRI techniques for mTBI studies with outcome and symptom associations described. The corpus callosum (CC) is one of the most studied fiber tracts in TBI and mTBI, but the comprehensive post-mTBI symptom relationship has not fully been explored.

**Methods:**

This is a retrospective observational study of how quantitative DTI data of the CC and its sub-regions may relate to clinical presentation of symptoms and timing of resolution of symptoms in patients diagnosed with uncomplicated mTBI. DTI and clinical data were obtained retrospectively from 446 (mean age 42 years, range 13–82) civilian patients. From patient medical charts, presentation of the following common post-concussive symptoms was noted: headache, balance issues, cognitive deficits, fatigue, anxiety, depression, and emotional lability. Also recorded was the time between injury and a visit to the physician when improvement or resolution of a particular symptom was reported. FA values from the total CC and 3 subregions of the CC (genu or anterior, mid body, and splenium or posterior) were obtained from hand tracing on the Olea Sphere v3.0 SP12 free-standing workstation. DTI data was obtained from 8 different 3T MRI scanners and harmonized via ComBat harmonization. The statistical models used to explore the association between regional Fractional Anisotropy (FA) values and symptom presentation and time to symptom resolution were logistic regression and interval-censored semi-parametric Cox proportional hazard models, respectively. Subgroups related to age and timing of first scan were also analyzed.

**Results:**

Patients with the highest FA in the total CC (*p* = 0.01), anterior CC (*p* < 0.01), and mid-body CC (*p* = 0.03), but not the posterior CC (*p* = 0.91) recovered faster from post-concussive cognitive deficits. Patients with the highest FA in the posterior CC recovered faster from depression (*p* = 0.04) and emotional lability (*p* = 0.01). There was no evidence that FA in the CC or any of its sub-regions was associated with symptom presentation or with time to resolution of headache, balance issues, fatigue, or anxiety. Patients with mTBI under 40 had higher FA in the CC and the anterior and mid-body subregions (but not the posterior subregion: *p* = 1.00) compared to patients 40 or over (*p* ≤ 0.01). There was no evidence for differences in symptom presentation based on loss of consciousness (LOC) or sex (*p* ≥ 0.18).

**Conclusion:**

This study suggests that FA of the CC has diagnostic and prognostic value for clinical assessment of mTBI in a large diverse civilian population, particularly in patients with cognitive symptoms.

## 1. Introduction

Over 1.7 million traumatic brain injuries (TBIs) are diagnosed in the United States every year (Menon et al., 2010). TBIs are classified clinically as either mild, moderate, or severe based primarily on the Glasgow Coma Scale score, post-traumatic loss of consciousness, or memory loss (Menon et al., 2010). Mild traumatic brain injuries (mTBIs), or concussions, are the most common type of TBI, representing approximately 80% of brain injuries in the US (Menon et al., 2010). The negative impacts of moderate and severe TBI are clinically and otherwise well described (Rutgers et al., 2008) but complications of an mTBI often persist for months or years after date of injury. For example, a large cohort study of 910 patients with mTBI reported that 6 months after the injury, 84% had post-traumatic complaints, 45% had emotional distress, and 44% had incomplete recovery based on the Glasgow Outcome Scale Extended (van der Naalt et al., 2017).

Difficulties relating to the diagnosis and treatment of mTBI can be in part attributed to disparate clinical presentation and diagnostic criteria (Gómez et al., 1996; Ruff et al., 1996; McCrory et al., 2013; Koerte et al., 2016; Kazl and Torres, 2019; Pozzato et al., 2020). At present an mTBI diagnosis is heavily reliant on physician assessment of patient clinical history. Anticipating the severity and longevity of post-traumatic sequalae remains challenging (Menon et al., 2010). Dynamic common post-concussive symptoms include somatic as well as neuropsychological complaints, including headache, dizziness/difficulty balancing, cognitive deficits, fatigue, lethargy, altered emotional state, and behavioral and personality changes (Tator, 2013; Kazl and Torres, 2019). A physician can diagnose post-concussion syndrome (PCS) after mTBI when symptoms persist 3 or more months based on the International Classification of Diseases, 10^th^ revision (Organization World Health, 1993). The diagnosis of PCS is somewhat controversial, and the most recent version of the Diagnostic and Statistical Manual of Mental Disorders [5^th^ edition; DSM-IV; criteria (American Psychiatric Association, 2013)] dropped PCS. The ICD-10 based diagnosis of PCS is made when 3 of 8 of the following symptoms are present: headache, dizziness, fatigue, irritability, insomnia, concentration difficulty, memory difficulties, or intolerance of stress, emotion, or alcohol. This is important for this study because many previous studies (Khong et al., 2016) have used PCS as an outcome variable, and since each of the individual symptoms may have a separate neurological basis, it is possible that the use of PCS as an outcome variable has obscured some useful neuroradiological associations with individual symptoms. Inaccurate or incomplete reporting of factors such as symptom severity, loss of consciousness, and post-traumatic amnesia may confound meaningful clinical assessment (Menon et al., 2010). To add to the complications, patients over 40 years old have slower recovery from mTBI compared to patients under 40 (Chiang et al., 2016).

There is considerable interest in expanding existing clinical diagnostic techniques with objective and quantitative parameters (Tator, 2013). However, image findings consistent with trauma are seldom detected on conventional magnetic resonance imaging (MRI) modalities, appearing <27% of the time for patients with mTBI (Yuh et al., 2013). Studies using a more advanced quantitative MR imaging technique, Diffusion tensor imaging (DTI), have suggested that trauma may induce subtle microstructural changes to the white matter not visible in CT or conventional MRI in some (Tisserand et al., 2006; Cubon et al., 2011; Lipton et al., 2012; Toth et al., 2013; Hayes et al., 2015; Bahrami et al., 2016), but not all (Ilvesmäki et al., 2014), studies. It appears clear that Diffusion Tensor Imaging (DTI) offers enhanced sensitivity to detect structural white matter changes in a variety of fiber tracts in mTBI patients with a variety of reported outcome and symptom associations (Narayana, 2017; Palacios et al., 2022).

DTI measures variable diffusion of water molecules to quantitatively assess the physical properties of white matter and estimate axonal health (Arfanakis et al., 2002; Hulkower et al., 2013). DTI makes the fundamental assumption that in a healthy neuron, the water diffusion signal along an axon is unconstrained relative to diffusion, and constrained perpendicular to the path of axonal projection. Diffusion data collected in DTI can be expressed in terms of four quantitative variables: fractional anisotropy (FA), mean diffusivity (MD), radial diffusivity (RD), and axial diffusivity (AD) (Arfanakis et al., 2002). FA values are most often used in clinical practice and the focus of research trials for a variety of neurologic conditions and disorders including TBI (Tae et al., 2018). Small FA values (near zero) suggest unconstrained diffusion in all directions, while large FA values (near one) are taken to indicate that diffusion is restricted in all directions except along one axis, although strict interpretation is not without controversy (Figley et al., 2022). While some studies have described elevated FA values in the acute phase and a minority in the chronic phase, the most widely reported finding in mTBI patients is decreased FA values, thought to represent impediment of water diffusivity along axons due to microscopic injury (Lindsey et al., 2021). A large recent 391 subject multicenter and scanner mTBI study found significantly decreased FA values as soon as 2 weeks following mTBI (Palacios et al., 2022). Recent diffusion imaging studies suggest that microstructural white matter changes can be detected in mTBI, and that decreased FA values are common among patients experiencing prolonged post-concussive symptoms (Gonzalez et al., 2021a; Ware et al., 2022). A complicating factor is age, which can also affect FA, where individuals over 40 years old have lower FA than younger individuals (Ilvesmäki et al., 2014).

**Figure 1:**
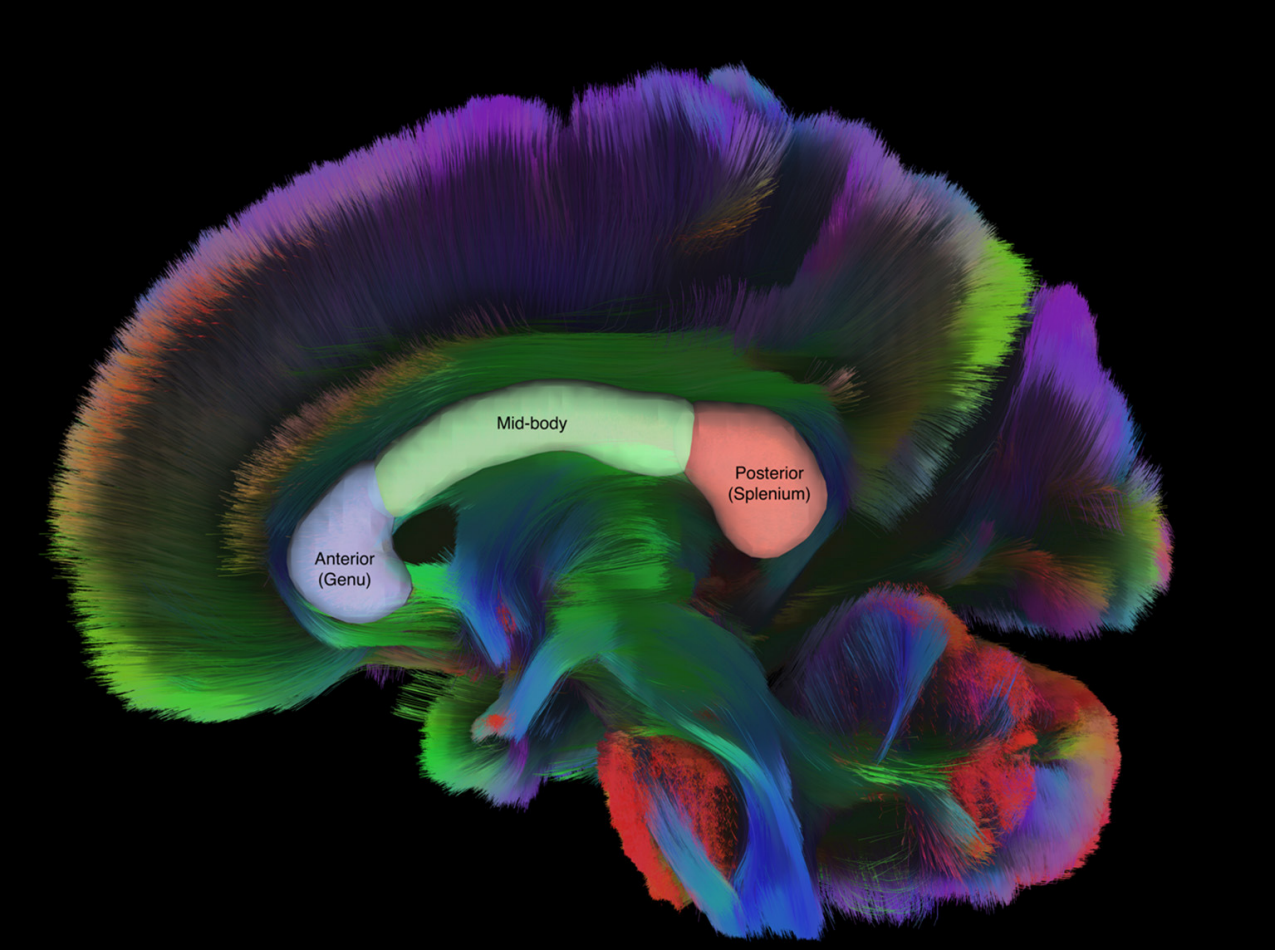
Sagittal tractography of the CC with labeled regions of interest drawn over representative sub regions of the CC. Figure generated in DSI Studio (v10.15).

The corpus callosum (CC) is an area of the brain that appears to be particularly important in evaluating effects of mTBI, based on its location, vulnerability to axonal shearing, and previous studies. The CC is the largest white matter structure of the brain, serving as the primary inter-hemispheric bridge. Anatomically, the CC can be described in terms of three sub-regions: the genu (anterior portion), mid-body (medial portion), and splenium (posterior portion; Figure 1) (Haines and Ard, 2013). Each sub-region has been related to its function by examining activation using fMRI (Fabri and Polonara, 2013); for example, the splenium is activated in response to stimuli that take and auditory or visual form, whereas the genu responds to taste and the central portion to motor tasks. Previous studies of mTBI have also used these sub-regions of the CC (Nakayama et al., 2006; Matsushita et al., 2011; Wallace et al., 2018).

The CC is of particular interest in TBI due to the structural and spatial vulnerability to axonal shearing injuries (King et al., 1995). Axonal damage outside the CC may propagate to the CC via Wallerian degeneration (Ho et al., 2013). The CC is one of the most well studied axonal tracts in TBI due to its critical location and large size, which has allowed a high level of technical reproducibility (Narayana, 2017; Lindsey et al., 2021). Multiple studies have found lower CC FA values in trauma patients vs. controls; however, many of these studies were relatively small or focused on the entire CC rather than its subregions (Rutgers et al., 2008; Kumar et al., 2009; Rajesh et al., 2017; Yin et al., 2019). Cognitive deficits are by far the most common symptom association with CC FA in trauma patients. A DTI meta-analysis including the CC in 562 TBI patients, of which 229 were mTBI patients, did not distinguish between symptom presentation and improvement and there was some heterogeneity in the cognitive assessments with 7 different domains categorized (Wallace et al., 2018). Other common symptoms following mTBI (e.g., headache, balance deficits, fatigue, anxiety, depression, and emotional lability) have been less well-studied. In addition, much of the current literature does not clearly distinguish between symptom presentation and symptom resolution or improvement, which is an important distinction for clinicians in making diagnostic and treatment decisions. Specifically, symptom resolution or improvement is assessed only for the subset of patients who present with a symptom, whereas associations with symptom appearance are made in comparison to those who do not have a symptom.

The purpose of this study was to examine FA of CC regions more comprehensively with comparison to symptom incidence and time to resolution for 7 of the most commonly reported mTBI symptoms. To our knowledge, this study of 466 mTBI patients is the largest study to date using DTI to assess mTBI patients. Previous studies have demonstrated that decreased fractional anisotropy (FA) of the CC after mTBI is often correlated with prolonged, post concussive symptomatology, particularly cognitive deficits. We hypothesized that isolating sub-regions of the CC and relating their FA to individual post-mTBI symptom presence and persistence will yield additional insights and testable hypotheses to inform future work.

## 2. Methods

### 2.1. Patient population

The patients included in the study were all in litigation. Patients with initial imaging up to 6 years post-injury were considered for inclusion. Imaging and clinical data were obtained retrospectively for 717 civilian patients who were diagnosed with mTBI by a board-certified neurologist specializing in head trauma utilizing the standard DSM-V diagnostic criteria (American Psychiatric Association, 2013). Neurology initial consult occurred 118 days post-injury on average and no subjects were reported in the record as having a Glasgow Coma Scale (GCS) of 14 or lower at the time of the trauma. All patients reported persistence of one or more injury related symptoms for longer than 6 months post-injury. Records which were incomplete, with canceled/incomplete imaging, or with initial imaging >6 years post-injury, were excluded from analysis. Patients with a clinical history of demyelinating disease, cerebrovascular accidents/infarcts, prior head trauma, malignant/benign brain tumors, chronic epilepsy, or brain surgery were excluded from the study. All imaging data was reviewed by two board-certified neuroradiologists to check for the presence of the following exclusionary findings: brain contusions, hemorrhagic diffuse axonal injury, prior intracranial hemorrhaging, cerebral vascular malformation, cavernoma, brain tumor, motion artifact, or other technical artifact (Vanier et al., 2020). Of the 717 patients screened in the database, 446 met the inclusion criteria and 402 (90% of the total) had scans conducted in the first year after injury. This study was determined exempt by the Touro University Nevada Institutional Review Board (IRB).

### 2.2. Clinical measures

Patient clinical data was obtained via retrospective neurologist chart review. Symptom presentation, progression and improvement of cognitive deficits, headache, fatigue, depression, anxiety, balance issues, and emotional lability were codified at each neurological consult, which occurred at non-regular intervals. Cognitive symptoms were defined broadly as any impairment in learning and memory, attention, executive functions and language communication (Silver et al., 2005). The cognitive symptoms were assessed by standard instruments (Jones et al., 2021) as well as by a neurologist. Headaches were based on self-reports. Balance was broadly defined by the neurologist based on physical examination and/or tests outlined by Lei-Rivera et al. (2013). Anxiety, depression, and emotional lability were diagnosed using standard DSM-V diagnostic criteria (American Psychiatric Association, 2013). Time between the injury and the first clinical consultation was noted. The longevity or survival time of a symptom was recorded using the two clinical consultations bracketing the date when clinical symptoms improved or resolved. To create an interval censored dataset, the number of days between injury and the two clinical consultations was recorded. In some individuals, the symptoms did not improve or resolve, so the final clinical consultation was noted, and the data was considered right-censored (Vanier et al., 2020).

### 2.3. Image acquisition and processing

DTI data was obtained using 8 different 3T MRI scanners (Table 1). Scanning parameters varied slightly but were all echo planar sequences capturing at least 33 directions and maximum slice thickness of 4 mm. 348 of the 446 datasets collected had a slice thickness of 2.6 mm or less. All diffusion imaging data was manually inspected for artifacts. The raw diffusion imaging data sets were then preprocessed and reconstructed using a commercially available Olea Sphere v3.0 SP12 free-standing workstation. Prior to reconstruction, raw diffusion images were motion and eddy-current corrected in Olea. A Fiber Assignment with Continuous Tracking (FACT) algorithm was also deployed via the proprietary Olea preprocessing workstation to minimize partial volume effects of crossing fibers and free water. After successful reconstruction was confirmed, an experienced technologist hand traced the regions of interest (ROI) containing white matter tracts while avoiding the nearby cerebrospinal fluid filled ventricles. These regions included the anterior CC (Genu), mid-body of the CC (Body), posterior CC (Splenium), and the total CC. The total CC included all three CC subregions. To maximize translatability of findings to clinical practice of neuroradiology, and also to maximize intercomparison between recently published studies, this study used FA values. After ROI selection, an average FA value was calculated for each of the four ROIs. These average FA values were then transcribed into the patient's medical record and were then recollected during the chart review process.

**Table 1:** Location and MRI information for scans included in the study.

**Location**	**Manufacturer**	**Model**	**Repetition time (ms)**	**In-plane resolution (mm)**	**Gradient directions (#)**	**Echo time (ms)**	**Number of patients**
1	GE	Signa HDxt	11,000	2 x 2 x 2.4	33	86	85
2	GE	Signa EXCITE	11,000	2 x 2 x 2.4	33	86	13
3	GE	Signa HDxt	11,000	2 x 2 x 2.4	33	85.1	125
4	Philips	Intera	4,302	2 x 2 x 2.2	33	81	87
5	Siemens	TrioTim	4,900	2 x 2 x 2.4	33	94	11
6	Siemens	Verio	4,200	2 x 2 x 4	33	95	114
7	Siemens	Verio	4,700	2 x 2 x 2.6	33	96	2
8	Siemens	Verio	4,700	2 x 2 x 2.6	33	96	9

All scanners employed in this study had a field strength of 3T and a standardized imaging protocol utilizing a flip angle of 90° and b-value of 1000.

### 2.4. Diffusion data harmonization

To minimize inter-scanner variability without altering intra-scanner reliability, data harmonization was performed using a modified ComBat harmonization technique. The ComBat data harmonization technique was first deployed in the context of gene expression microarrays (Johnson et al., 2007). The ComBat technique has proven effective at minimizing MRI scanner and site effects while still preserving biological variability due to injury (Fortin et al., 2017; Beer et al., 2020; Onicas et al., 2022). This study implemented the ComBat harmonization technique first by categorizing each patient's ROI average FA data set by scanner. The ComBat algorithm outlined in Fortin et al., 2017 in MATLAB 2020 was then modified to accept these datasets and then the algorithm was run.

### 2.5. Statistical analysis

This is a retrospective study which relies on observational clinical data. The analysis focused on describing the data relative to potential mediators of mTBI outcomes or DTI observations, characterizing prevalence of symptoms and their improvement during the study, then relating the FA of the entire CC and its sub-regions to the presence and persistence of the seven symptoms available in the patient medical charts. To understand differences in potentially relevant participant sub-populations, comparisons of mean FA for male/female, loss of consciousness/no loss of consciousness, and age <40/≥40 yrs were completed via *t*-test, and *p*-values were Bonferroni adjusted to control per-family error rates within each null hypothesis (Rubin, 2017; Frane, 2021). The relationship between regional CC FA values and presentation of post-concussive symptomatology was modeled using logistic regression (with symptom presence or absence as dependent variable and FA as a continuous independent variable), and the coefficient and standard error were used to compute the odds (and unadjusted 95% confidence interval) of presenting with a symptom with a 0.01 unit increase in FA. A change of 0.01 represents ~4–5% of the range in the FA data set (**Table 3**). The logistic regressions were also done on subgroups based on age (<40, ≥40) to check sensitivity of the findings to age using a breakpoint associated with poorer mTBI recovery and FA.

The association between CC FA values and symptom persistence was assessed using an interval-censored semi-parametric Cox proportional hazard model, and (unadjusted) *p*-values were generated via an exact log-rank trend test with 999 Monte Carlo replications. Log-rank scores were used to assess directionality of trends such that positive scores indicate a positive correlation between larger regional FA values and faster improvement or resolution of symptoms (Fay, 1999). In the proportional hazard models, FA was treated as a continuous variable. To provide more insight to relative effect size, Kaplan-Meier curves are presented to characterize the symptom longevity for patients with the FA categorized into tertiles: the upper third of FA, middle third of FA, and lowest third of FA, by sub-region. To detect sensitivity to time between date of injury and scan, sensitivity analyses of FA relative to symptom presence and longevity were performed on the subset of 402 (90% of the total) patients with scans conducted in the first year after injury. R software (v4.0.5) and the “interval” package were used to complete the data analysis (Fay and Shaw, 2010; Team, 2020).

## 3. Results

The age of the 446 patients included in the study ranged from 13 to 82, with median age of 42; 59% were female (Table 2). The median time between scanning and injury was 100 days (range 6 to 1,854 days; Table 2). Most (81%) patients were injured in motor vehicle collisions. The two sexes lost consciousness at approximately the same rate (males: 56 lost consciousness of 184; females: 60 of 262; Fisher exact test *p* = 0.49), and were represented equally in age groups <40 (112 females, 73 males) and those ≥40 (150 females, 111 males; *p* = 0.56). Patients <40 (55 lost consciousness of 185) lost consciousness at approximately the same rate as those ≥40 (61 lost consciousness of 261; *p* = 0.15).

**Table 2:** Subject demographics and details of injury by gender.

	**Female**			**Male**			**Total**		
*n*		262	59%		184	41%		446	100%
**Cause of injury**									
Assault		4	2%		9	5%		13	3%
Fall		23	9%		14	8%		37	8%
Falling object		12	5%		7	4%		19	4%
Miscellaneous		6	2%		4	2%		10	2%
Motor vehicle collision		214	82%		148	80%		362	81%
Unknown		3	1%		2	1%		5	1%
LOC at time of TBI		60	23%		56	30%		116	26%
		IQR	Range		IQR	Range		IQR	Range
Age	42	(29, 53)	(13, 82)	43	(32, 54)	(16, 80)	42	(31, 53)	(13, 82)
Days from injury to scan	93	(50, 175)	(6, 1210)	110	(56, 180)	(14, 1854)	100	(52, 179)	(6, 1854)

*n*, sample size; LOC, Loss of Consciousness. Median, interquartile range (IQR), and range are reported for age and days of injury to scan.

The mean FA (standard error; minimum-maximum) for the total CC was 0.595 (0.001, 0.503–0.698), for the Anterior CC was 0.557 (0.001, 0.484–0.671), for the Mid-Body CC was 0.584 (0.002, 0.450–0.725), for the Posterior CC was 0.637 (0.001, 0.463–0.737). There were no overall differences in mean FA between female and male patients or between those who did and did not report LOC (Table 3). Patients <40 had higher mean FA for the whole CC and its anterior and mid-body sub-regions compared to patients ≥40 (Table 3). There was no statistical support for a difference between the two age groups for mean FA in the posterior CC (Table 3). In support of the decision to analyze sub-regions of the CC separately, the FA in different regions of the CC were moderately correlated, with Pearson correlations between 0.35 (anterior CC to posterior CC) and 0.50 (mid-body CC to anterior CC; Supplementary Table S1). FA values of the total CC were most strongly correlated with the posterior and mid-body regions (*r* = 0.62 with posterior CC, *r* =0.73 with mid-body CC) which is generally consistent with the relative size of these regions to the total CC (Supplementary Table S1).

**Table 3:** Summary of FA by CC region.

	**Mean FA**	**SE**	**Minimum**	**Maximum**		**Range**
Total	0.595	0.001	0.503	0.698		0.195
Anterior	0.557	0.001	0.484	0.671		0.187
Mid-body	0.584	0.002	0.450	0.725		0.275
Posterior	0.637	0.001	0.463	0.737		0.274
	**Female**	**Male**	**SE**	***t***	**df**	**Adjusted** ***p***
Total	0.593	0.598	0.003	−1.9	389	0.26
Anterior	0.556	0.558	0.003	−0.9	409	1.00
Mid−body	0.582	0.586	0.003	−1.4	417	0.64
Posterior	0.635	0.640	0.003	−1.5	395	0.52
	**No LOC**	**With LOC**	**SE**	***t***	**df**	**Adjusted** ***p***
Total	0.594	0.599	0.003	−1.7	189	0.38
Anterior	0.555	0.561	0.003	−1.7	201	0.36
Mid−body	0.582	0.589	0.004	−2.0	196	0.18
Posterior	0.636	0.639	0.003	−0.8	194	1.00
	<**40**	≥**40**	**SE**	***t***	**df**	**Adjusted** ***p***
Total	0.599	0.592	0.002	3.0	404	**0.01**
Anterior	0.568	0.549	0.003	6.9	389	**< 0.01**
Mid-body	0.589	0.580	0.003	2.9	406	**0.01**
Posterior	0.639	0.636	0.003	1.1	427	1.00

Mean and pooled standard error (SE) FA values for sex, loss of consciousness (LOC), and age group. The *t* statistic, degrees of freedom to test group differences, and Bonferroni adjusted (within each null hypothesis) *p*-values are also reported. Adjusted *p*-values below 0.05 are in bold.

The most common symptoms patients reported during initial clinical consultation were headache (94%), balance problems (70%), and cognitive deficits (73%; Table 4). The most frequently reported symptoms also had the highest rate of improvement, with 41%−43% of patients reporting resolution or improvement at some point during clinical consultation. Anxiety (28%) and depression (24%) were less frequently reported, and less likely to improve (20% and 23% respectively) while fatigue (17%) and emotional lability (15%) were the least common symptoms and also unlikely to improve (17% and 20%, respectively).

**Table 4:** Prevalence of symptoms and symptom resolution or improvement by number of patients.

	**Presentation**	**Improvement**
Headache	420 (94%)	178 (42%)
Balance problems	310 (70%)	133 (43%)
Cognitive deficits	325 (73%)	133 (41%)
Fatigue	75 (17%)	13 (17%)
Anxiety	123 (28%)	25 (20%)
Depression	105 (24%)	24 (23%)
Emotional lability	66 (15%)	13 (20%)

The percent occurrence was based on the entire patient population, whereas the percent for improvement or resolution was based on the number of affected patients for within each symptom.

Across all patients, there was no evidence that FA of the entire CC or its sub-regions was related to the presence of any of the seven clinically assessed post-concussive symptoms (Table 5). When age groups were examined separately, there was an association between low FA in the posterior CC and balance problems in patients aged 40 and over (Supplementary Table S2).

**Table 5:** Symptom presence relative to FA of corpus callosum regions.

**Symptom**	**CC region**	**Estimate**	**SE**	**Odds**	**95% Confidence interval**
**Headache**					
	Total	0.07	0.08	1.07	0.92, 1.24
	Anterior	0.02	0.07	1.02	0.89, 1.17
	Mid-body	0.03	0.06	1.03	0.92, 1.16
	Posterior	−0.04	0.07	0.96	0.84, 1.09
**Balance**					
	Total	−0.06	0.04	0.94	0.87, 1.02
	Anterior	−0.07	0.04	0.93	0.87, 1.00
	Mid-body	−0.04	0.03	0.96	0.90, 1.02
	Posterior	−0.07	0.03	0.93	0.87, 1.00
**Cognitive**					
	Total	−0.03	0.04	0.97	0.89, 1.05
	Anterior	−0.05	0.04	0.95	0.88, 1.02
	Mid-body	−0.01	0.03	0.99	0.93, 1.05
	Posterior	−0.02	0.03	0.98	0.92, 1.05
**Fatigue**					
	Total	−0.03	0.05	0.97	0.89, 1.07
	Anterior	−0.05	0.04	0.95	0.87, 1.04
	Mid-body	−0.02	0.04	0.98	0.91, 1.05
	Posterior	−0.01	0.04	0.99	0.91, 1.07
**Anxiety**					
	Total	0.01	0.04	1.01	0.94, 1.10
	Anterior	0.02	0.04	1.02	0.95, 1.09
	Mid-body	0.01	0.03	1.01	0.96, 1.08
	Posterior	0.01	0.03	1.01	0.94, 1.08
**Depression**					
	Total	0.00	0.04	1.00	0.92, 1.09
	Anterior	0.02	0.04	1.02	0.94, 1.09
	Mid-body	−0.02	0.03	0.98	0.92, 1.05
	Posterior	−0.02	0.04	0.98	0.92, 1.05
**Emotional lability**					
	Total	−0.05	0.05	0.95	0.86, 1.05
	Anterior	−0.02	0.05	0.98	0.89, 1.07
	Mid-body	−0.02	0.04	0.98	0.91, 1.06
	Posterior	−0.01	0.04	0.99	0.91, 1.08

The logistic regression coefficient (estimate) and its standard error (SE) are shown, and the change in odds (and 95% confidence interval) of presenting with a symptom associated with a 0.01 unit increase in CC region FA is also provided.

**Figure 2:**
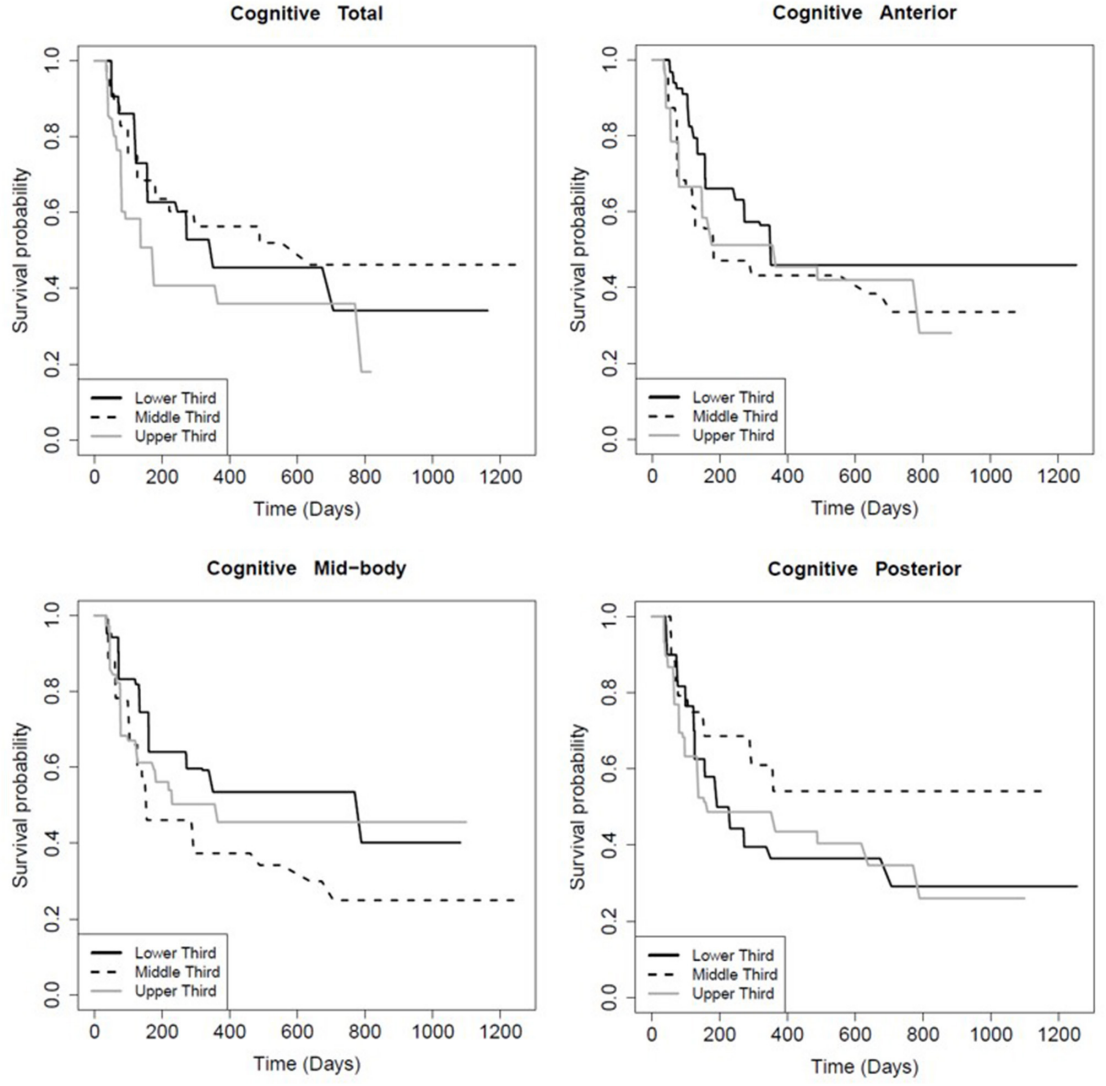
Kaplan-Meier survival curve showing the longevity of post-concussive cognitive deficits for patients in the upper (highest FA), middle, and lower (smallest FA) third of CC FA values, total and by region as indicated. The Y-axis is the percent of patients reporting symptom persistence at a given time point post-injury. Supporting statistics in Table 5.

**Figure 3:**
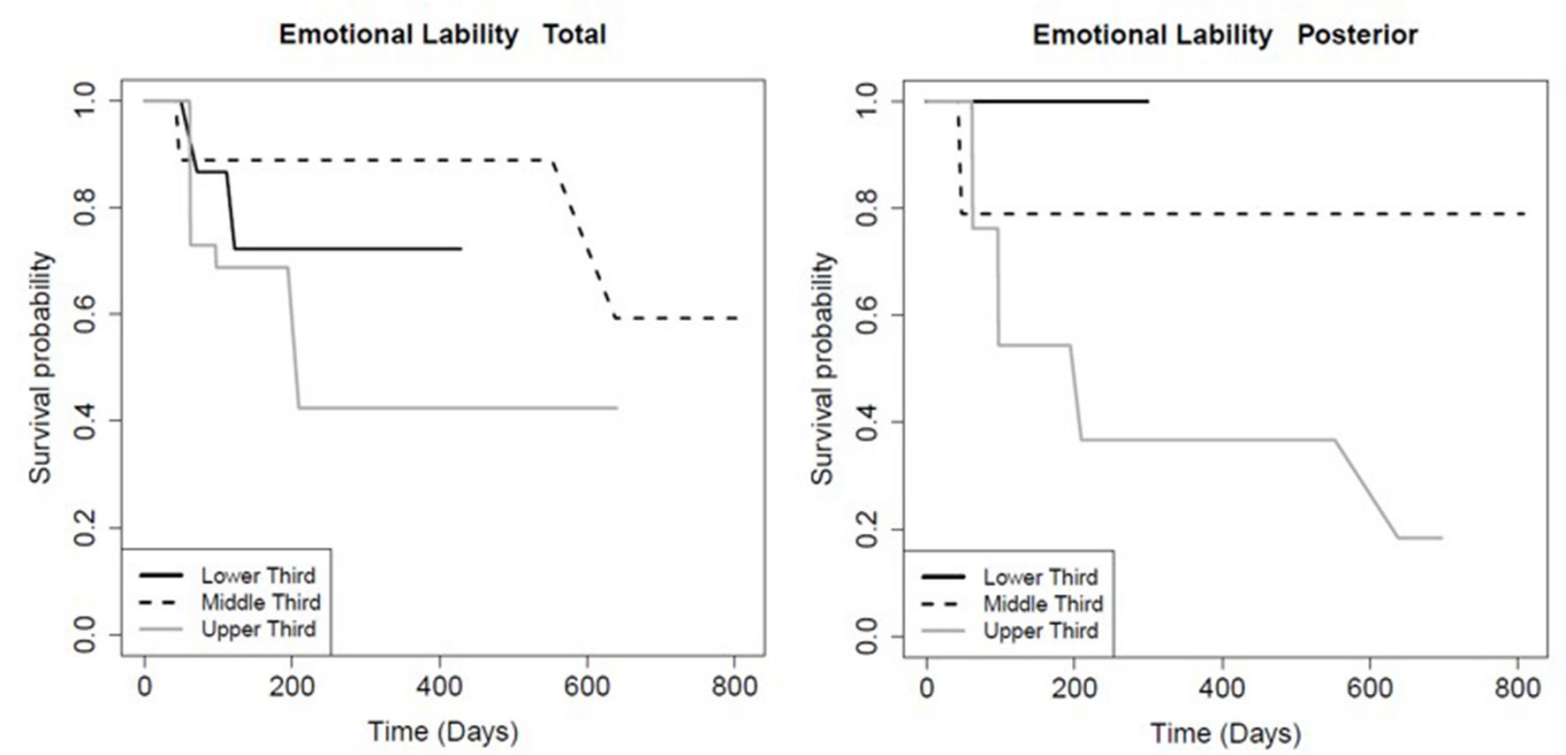
Kaplan-Meier survival curve showing the longevity of post-concussive emotional lability for patients in the upper (highest FA), middle, and lower (smallest FA) third for total and posterior CC FA, as indicated. Details as in Figure 2.

**Figure 4:**
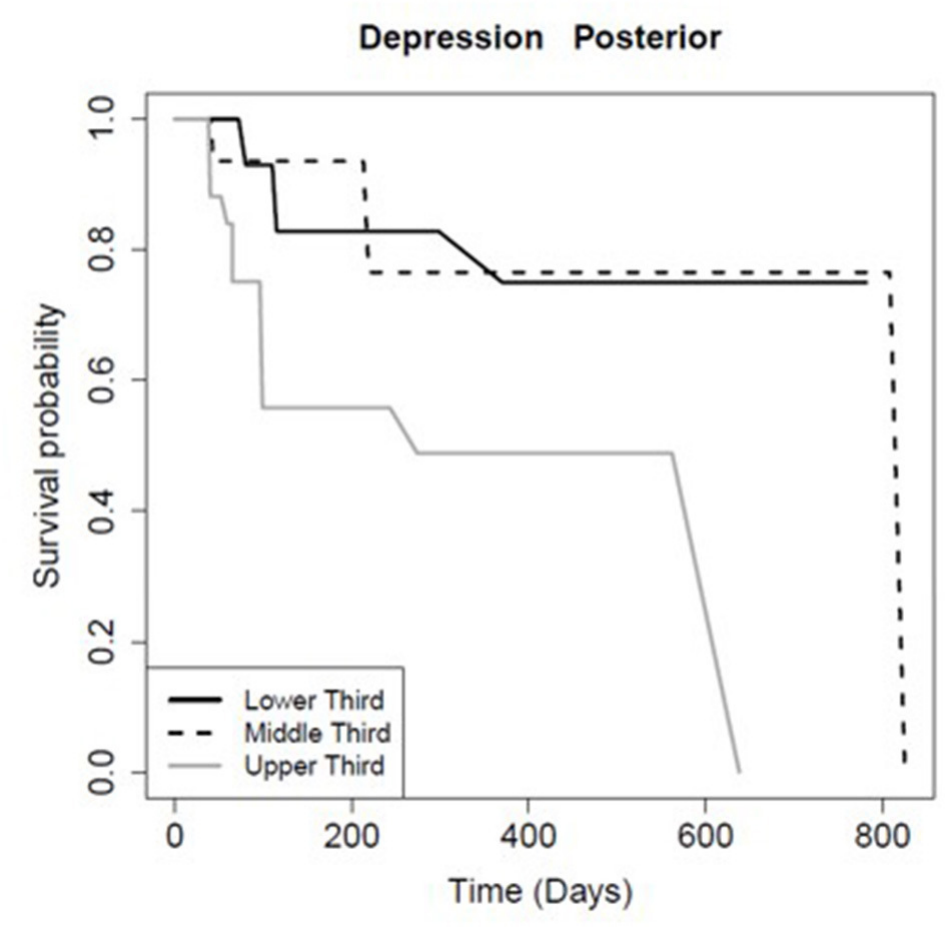
Kaplan-Meier survival curve showing the longevity of post-concussive depression for patients in the upper (highest FA), middle, and lower (smallest FA) third for posterior CC FA, as Figure 2.

The FA of the entire CC and its sub-regions had prognostic value for persistence of certain symptoms. Patients with FA values in the higher tertile for the total CC (*p* = 0.01), mid-body CC (*p* = 0.03), and anterior CC (*p* < 0.01) recovered significantly faster from post-concussive cognitive deficits relative to the lowest tertile (Table 6; Figure 2). There was no association between FA in the posterior CC and longevity of cognitive symptoms (*p* = 0.91; Table 6, Figure 2). However, patients with FA in the posterior CC that was in the lower tertile had slower symptom improvement or resolution compared to the highest FA tertile for post concussive depression (*p* = 0.04) and emotional lability (*p* = 0.01; Table 6; Figures 3, 4). There was no statistical evidence that FA of the CC or its regions predicted the longevity of headache, balance, fatigue, or anxiety symptoms (Table 6).

**Table 6:** Symptom longevity relative to FA of corpus callosum regions.

					**Percent participants with resolved symptoms**					
					**6 months after injury**			**12 months after injury**		
**Symptom**	***p*** **-value**	***p*** **-value**	**CI**	**Score Statistic**	**Lower 1/3**	**Middle 1/3**	**Upper 1/3**	**Lower 1/3**	**Middle 1/3**	**Upper 1/3**
**Headache (*****n** =* **420)**										
Total	0.83	0.75	0.91	0.08	48	45	47	64	52	55
Anterior	0.43	0.36	0.49	0.29	45	47	46	59	58	54
Mid-body	0.50	0.43	0.57	0.30	39	61	45	57	63	51
Posterior	0.74	0.66	0.82	0.14	44	50	50	63	54	55
**Balance (*****n** =* **310)**										
Total	0.56	0.49	0.64	−0.16	47	48	42	66	52	49
Anterior	0.90	0.81	0.98	−0.07	41	54	40	59	62	45
Mid-body	0.42	0.35	0.49	0.30	38	54	47	55	69	54
Posterior	0.45	0.39	0.52	0.28	46	47	46	60	54	51
**Cognitive (*****n** =* **325)**										
Total	**0.01**	**< 0.01**	**0.03**	**0.73**	**37**	**36**	**59**	**54**	**44**	**64**
Anterior	**< 0.01**	**< 0.01**	**0.01**	**0.95**	**34**	**53**	**49**	**54**	**57**	**55**
Mid-body	**0.03**	**0.02**	**0.06**	**0.77**	**36**	**54**	**44**	**47**	**63**	**54**
Posterior	0.91	0.83	0.99	0.05	42	31	51	64	46	56
**Fatigue (*****n** =* **75)**										
Total	0.10	0.07	0.14	0.13						
Anterior	0.50	0.43	0.57	0.06						
Mid-body	0.86	0.78	0.94	0.01						
Posterior	0.07	0.04	0.11	0.20						
**Anxiety (*****n** =* **123)**										
Total	0.14	0.10	0.19	0.18						
Anterior	0.53	0.46	0.60	0.10						
Mid-body	0.14	0.10	0.18	0.23						
Posterior	0.15	0.11	0.20	0.20						
**Depression (*****n** =* **105)**										
Total	0.17	0.13	0.22	0.17						
Anterior	0.20	0.16	0.26	0.17						
Mid-body	0.35	0.29	0.42	0.16						
Posterior	**0.04**	**0.02**	**0.06**	**0.29**						
**Emotional lability (*****n** =* **66)**										
Total	0.09	0.06	0.13	0.15						
Anterior	0.18	0.13	0.23	0.13						
Mid-body	0.11	0.07	0.15	0.19						
Posterior	**0.01**	**< 0.01**	**0.03**	**0.26**						

The permutation-based *p*-value and its 95% confidence interval (ci), and log rank score statistics are provided for each combination of region and symptom. The rows with a *p*-value < 0.05 are in bold. *P*-values were not adjusted for multiple comparisons. For tertiles with sample size >15, the percent of participants with resolved symptoms is reported at 6 months and 12 months, based on Turnbull intervals.

Sensitivity analyses of FA of the CC relative to symptom presence/longevity on a subset (*n* = 402) of patients yielded the same conclusions as analyses involving the full data set (Supplementary Tables S3, S4). The most notable exception was that the relationship between posterior CC FA and longevity of depression symptoms had weaker statistical support (*p* = 0.04 with the full data set compared to *p* = 0.09 for the 1-year scan subset), although the score statistics were approximately the same in magnitude (0.24 with the full data set compared to 0.29 for the 1-year scan subset; Table 6, Supplementary Table S4).

## 4. Discussion

This study is concordant with others reporting significant associations between mTBI and white matter diffusion properties of the CC (Jokinen et al., 2007; Aoki et al., 2012; Iraji et al., 2016; Khong et al., 2016). FA in the CC was not related to the presence of particular mTBI symptoms. The FA in the CC was related to the resolution or improvement of some of the common mTBI symptoms, such as cognitive deficits, depression, and emotional lability. Despite the large sample size and associated high statistical power relative to other studies, this study found no evidence that FA of the CC or its sub-regions can, when used alone, provide prognostic information for persistence of headache, balance problems, fatigue, or anxiety in a population of people who have experienced mTBI. This result does not exclude the use of FA of the CC as part of a multivariate data set to improve predictive power (e.g., Iraji et al., 2016).

### 4.1. Age

Given the lower mean FA in patients ≥40 compared to those <40, there is the potential of confounding between age and FA, particularly in light of notable parallels between this study (looking at FA in the CC) and previous work involving a subset of the patients in this study (250 mTBI patients) (Vanier et al., 2020). Vanier et al. (2020) found associations between age and symptom presence and persistence and found that older patients (≥40) were more likely to present with balance symptoms. In this study, presentation of balance problems was related to low FA in the posterior CC, only within the group of patients ≥40. The low FA in the posterior CC (splenium) after mTBI may therefore provide additional indications regarding balance symptom presentation in patients ≥40. For symptom persistence, in the previous study, cognitive deficits in patients ≥40 were slower to improve or resolve. In this study, patients with lower FA in the total/anterior/mid-body CC had more persistent cognitive symptoms. The expected parallels between the two studies is based on previous studies, which report lower FA (Ilvesmäki et al., 2014) and slower recovery from mTBI (Chiang et al., 2016) in older participants. The parallel between the two study results is not perfect; there are also some symptoms for which age-related patterns and FA-related patterns were not similar. For example, patients ≥40 had longer persistence of headache in Vanier et al., but this study found no relationship between headache persistence and FA in the CC.

The FA in the CC appeared in some cases to augment the prognostic information provided by age. It remains to be tested whether age-related delays in recovering from mTBI may be at least partly driven by FA in the CC or its sub-regions. This is a tentative conclusion based on indirect evidence, as it was not possible to directly address the hypothesis in the statistical analysis of symptom persistence, given the sample sizes that resulted from splitting the data set into age groups and the relatively large number of unimproved or unresolved cases. Within extant literature, age has been associated with prolonged recovery time post-mTBI (Katz et al., 2004; Chiang et al., 2016; Erlebach et al., 2017). Further study is required to separate age-related differences in symptom appearance and persistence from the less established association between FA in the CC and symptom appearance and persistence. Chronic medical conditions are known to impact FA values, with greater prevalence of these conditions in the elderly. Certain pathologies, such as diabetes and hypertension, may be present among patients studied here (Hannawi et al., 2018; Huang et al., 2021).

### 4.2. Cognition

This study corroborates the results of others in reporting an association between low FA in anterior/mid-body sub-regions and the persistence of post-concussive cognitive symptoms in TBI (Wallace et al., 2018). In contrast, there were not significant associations between decreased FA and presentation of cognitive symptoms, as reported elsewhere (Wallace et al., 2018). The cognitive diagnostics included in this study used the broadest available definition of cognitive problems, so results can only be qualitatively compared to, for example, a meta-analysis identifying CC DTI associations in all 7 cognitive domain subsets (Wallace et al., 2018).

The posterior CC (splenium) has been well-described as a white matter region that is vulnerable to trauma (Rutgers et al., 2008). Other studies have identified significant associations between cognitive symptomatology and reduced FA in the splenium (Nakayama et al., 2006; Matsushita et al., 2011); this study did not. An important distinction between this study and others is that this is a longitudinal study which consisted entirely of mTBI patients, while the other studies compared patients who experienced mTBI to those who had not. It is therefore possible that low FA in posterior CC is associated with mTBI, but not specifically with cognitive symptoms.

### 4.3. Headache, balance

The appearance or persistence of several of the symptoms considered in this study were not correlated with FA in the CC or its subregions. This overall finding is similar to the results reported by Lange et al., who found no differences in FA between groups of mTBI-affected individuals who did (*n* = 20) or did not (*n* = 52) have post-concussion syndrome as defined by ICD-10 criteria (Lange et al., 2015).

Similar to cognitive symptoms, there are other symptoms which are multifaceted and may not easily be simplified to one variable as in the current study. Headache is a very common complaint after mTBI, but this study found no evidence that headache appearance or persistence were correlated with FA of the CC or its sub-regions. This result is not in agreement with another study which compared mTBI-affected patients with (*n* = 58) and without (*n* = 17) migraines (Alhilali et al., 2017). They reported lower FA in the CC for patients with migraine, and they found a positive relationship between CC FA and time to recovery from headache. The disparity between Alhilali et al. (2017) and this study may be related to their focus on migraine headaches as more strictly defined by the international headache society which they distinguished from post concussive headaches and other headaches.

There do not appear to be any other studies that report any findings related to FA in the CC studying the specific symptom of balance/dizziness, other than the inclusion of dizziness in the list of possible symptoms of PCS. In this study, we found no evidence to support a link between FA in the CC and presence or persistence of balance problems except in the subgroup of patients ≥40 years old in the posterior CC.

### 4.4. Depression, anxiety, emotional lability, fatigue

Lower FA values in the posterior CC of patients with mTBI were predictive of persistent depression post-mTBI. Post-concussive depression is well-documented in the mTBI literature (Deb et al., 1999; McMahon et al., 2014), but has not been previously linked to changes in white matter diffusion as measured in DTI. There has been previous work linking post-mTBI depression and volume in the posterior CC; based on 435 persons with depressed behaviors, reduced volume of the posterior CC was associated with suicidal attempts compared to controls (Cyprien et al., 2011).

This study also found an association between FA values in the posterior CC and persistence of post-mTBI emotional lability. Emotional lability, while rare in the aftermath of mTBI, has been previously described in a pediatric population (Vasa et al., 2015). However, there appears to be a vacuum in the literature regarding how quantitative imaging may relate to post-concussive emotional changes. There is theoretical support for CC function with emotional lability. For example, a review of DTI studies in patients with borderline personality disorder, a condition in which emotional lability is a well-documented feature, found lower FA values in the CC (Sagarwala and Ha, 2020).

There do not appear to be any other studies that report any findings related to FA in the CC and the specific symptom of fatigue or anxiety in mTBI patients beyond the inclusion of related symptoms in the diagnosis of PCS. However, a recent review of DTI correlates of psychiatric conditions suggested that reduced FA in the CC could be functionally related to anxiety-related disorders (Podwalski et al., 2021).

### 4.5. Date of scan after injury

Most of the existing literature includes individuals who were scanned 12 months or less after injury, although there is one retrospective study which included patients whose mTBI was between one month to 45 years before the scan (Gonzalez et al., 2021b). It was therefore a possibility that date of scan relative to date of injury could impact the relationship between symptom presence or longevity and FA of the CC. In the sensitivity analysis to test this possibility, there was no evidence that including scans more than 12 months after the injury altered the findings of this study. Although damage from TBI is generally considered to be permanent, there is some evidence that FA may partially improve in the early chronic phase of mTBI, with corresponding improvements in functional outcomes (Lindsey et al., 2021).

## 5. Study limitations

The interpretation of the findings is limited by the retrospective observational study design, which is dependent on regular patient visits to assess symptom status. It is a strength of the study that a relatively large population of mTBI patients were included, which allows for heterogeneity within the injured population. The inclusion criteria make it impossible to know if the findings would also be relevant to populations of patients who do not have mTBI. The patients in the study were in litigation, so there may have been some motivation to report prolonged experience of symptoms and possibly impact mechanism. Cognitive symptoms were defined broadly and not subcategorized into any of the 7 cognitive domains (Wallace et al., 2018), nor were other multifaceted outcomes, such as headache, subcategorized. To avoid excessive right-censoring of clinical data, no differentiation was made between symptom resolution and improvement when defining symptom survival time.

As discussed above, the primary limitation of this study is that both DTI metrics and clinical outcomes in mTBI are known to be age-dependent (Jacobs et al., 2010; Sexton et al., 2014; Yue et al., 2019). It cannot be ruled out that changes in FA due to age or the comparatively increased rate of chronic disease in the elderly played a role in the results. However, while known to affect diffusion metrics, age has also been shown to function as an independent predictor of poor clinical outcomes post-mTBI (Vanier et al., 2020). Additional case control studies are needed to assess the extent to which diffusion metrics differentially predict outcomes in mTBI between older and younger patient populations. Finally, our image acquisition and processing protocol, manufacturer-specific differences in imaging equipment, and inherent subject-level neuroanatomic variation may impact FA values. Crossing of white matter fibers, while known to impact diffusion metrics, should not produce significant effects in DTI of the CC alone (Figley et al., 2022). Lastly, the ComBat harmonization of DTI parameters, while well-supported by the literature, is not without controversy, and diffusion metrics may differ if processed with an alternate harmonization technique (i.e., residuals and phenotype-adjusted residuals). Lastly, mechanism of injury and force direction were not analyzed.

## 6. Conclusion

In a large, diverse mTBI civilian population assessed for 7 common distinct TBI symptoms, higher FA values predicted improved outcomes in patients presenting with post concussive cognitive defects, depression, and emotional lability. In patients with cognitive symptoms this association was present in the anterior, mid-body and total CC sub-regions, but not the posterior CC. This study adds to the growing body of scientific literature highlighting ability of DTI to supplement existing clinical assessments of mTBI symptomatology, particularly in the area of cognitive deficits.

## Data availability statement

The original contributions presented in the study are included in the article/Supplementary material, further inquiries can be directed to the corresponding authors.

## Ethics statement

The studies involving humans were approved by Touro University Nevada College of Medicine IRB. The studies were conducted in accordance with the local legislation and institutional requirements. Written informed consent for participation was not required from the participants or the participants' legal guardians/next of kin in accordance with the national legislation and institutional requirements.

## Author contributions

AA, TK, AR, SP, TS, LG, and EF contributed to conception and design of the study. Clinical symptomatic data was transcribed and codified by TK, CL, BB, ME, RG, LZ, and AN. Diffusion imaging data was processed and harmonized by AA. AA, TK, and AR cleaned and organized combined imaging/clinical datasets. CV performed the statistical analysis. AA and TK wrote the first draft of the manuscript. CV, SA, and TS wrote sections of the manuscript. All authors contributed to manuscript revision, read, and approved the submitted version.
