# Supplementary material for: Diffusion in the corpus callosum predicts persistence of clinical symptoms after mild traumatic brain injury, a multi-scanner study
Source: Front Neuroimaging. 2023 Oct 31;2:1153115. doi: 10.3389/fnimg.2023.1153115 (PMC10654678; doi:10.3389/fnimg.2023.1153115)
Supplement: Supplementary file 1 [file Table_1.docx]

Supplementary Material

Article Title

A Asturias^1^, T Knoblauch^2,3^, A Rodriguez^2^, C Vanier^1^, C Le Tohic^3^, B Barrett^3^, M Eisenberg^3^, R Gibbert^3^, L Zimmerman^5^, S Parikh^2,4^, A Nguyen^5^, S Azad^5^, L Germin^7^, E Fazzini^1^, T Snyder^1,2,5,6^

**Correspondence:** Melinda Thrasher; Melinda.thrasher@imgen-research.com

# Supplementary Tables

Table S1. Pearson correlations for FA of total CC and CC subregions.

|  | total_cc | ant | mid | post |
| --- | --- | --- | --- | --- |
| total_cc | 1.00 |  |  |  |
| ant | 0.72 | 1.00 |  |  |
| mid | 0.73 | 0.50 | 1.00 |  |
| post | 0.62 | 0.35 | 0.48 | 1.00 |

Table S2. Data from Table 5, separated by age group. Symptom presence relative to FA of corpus callosum regions. The logistic regression coefficient (estimate) and its standard error (SE) are shown. The chi-squared statistic and associated p-value and odds ratio (OR) are also provided.

| **CC Region** | **Age <40** | | | | | **Age >40** | | | | |
| --- | --- | --- | --- | --- | --- | --- | --- | --- | --- | --- |
|  | **Estimate** | **SE** | **X^2^** | **p** | **OR** | **Estimate** | **SE** | **X^2^** | **p** | **OR** |
|  | Headache | |  |  |  | Headache | |  |  |  |
| Total | 13.9 | 15.5 | 0.8 | 0.377 | 1125569.85 | 1.7 | 8.9 | 0.0 | 0.844 | 5.71 |
| Anterior | 0.4 | 14.7 | 0.0 | 0.979 | 1.47 | -3.0 | 8.3 | 0.1 | 0.721 | 0.05 |
| Mid-body | 13.5 | 12.2 | 1.2 | 0.273 | 753451.55 | -2.2 | 6.8 | 0.1 | 0.746 | 0.11 |
| Posterior | 6.9 | 14.5 | 0.2 | 0.635 | 1033.12 | -8.0 | 7.7 | 1.1 | 0.288 | 0.00 |
|  | Balance |  |  |  |  | Balance |  |  |  |  |
| Total | -5.8 | 6.2 | 0.9 | 0.346 | 0.00 | -4.3 | 5.3 | 0.7 | 0.415 | 0.01 |
| Anterior | -5.9 | 5.5 | 1.2 | 0.279 | 0.00 | -6.5 | 5.0 | 1.7 | 0.193 | 0.00 |
| Mid-body | -2.9 | 4.7 | 0.4 | 0.542 | 0.06 | -4.1 | 4.1 | 1.0 | 0.310 | 0.02 |
| Posterior | -1.6 | 5.5 | 0.1 | 0.775 | 0.21 | **-10.7** | **4.7** | **5.6** | **0.018** | **0.00** |
|  | Cognitive | |  |  |  | Cognitive | |  |  |  |
| Total | -3.5 | 6.5 | 0.3 | 0.586 | 0.03 | -3.0 | 5.4 | 0.3 | 0.583 | 0.05 |
| Anterior | -2.7 | 5.7 | 0.2 | 0.634 | 0.06 | -6.9 | 5.1 | 1.8 | 0.178 | 0.00 |
| Mid-body | -2.3 | 4.9 | 0.2 | 0.643 | 0.10 | 0.2 | 4.1 | 0.0 | 0.963 | 1.21 |
| Posterior | 4.9 | 5.8 | 0.7 | 0.394 | 138.16 | -5.3 | 4.5 | 1.4 | 0.234 | 0.01 |
|  | Fatigue |  |  |  |  | Fatigue |  |  |  |  |
| Total | -2.2 | 7.9 | 0.1 | 0.786 | 0.12 | -2.5 | 6.2 | 0.2 | 0.682 | 0.08 |
| Anterior | -3.3 | 7.2 | 0.2 | 0.647 | 0.04 | -5.5 | 6.1 | 0.8 | 0.364 | 0.00 |
| Mid-body | 3.2 | 6.1 | 0.3 | 0.597 | 25.45 | -5.0 | 4.7 | 1.1 | 0.288 | 0.01 |
| Posterior | -1.5 | 7.2 | 0.0 | 0.830 | 0.21 | -1.2 | 4.9 | 0.1 | 0.805 | 0.29 |
|  | Anxiety |  |  |  |  | Anxiety |  |  |  |  |
| Total | 1.5 | 6.5 | 0.1 | 0.816 | 4.60 | 1.4 | 5.3 | 0.1 | 0.796 | 3.92 |
| Anterior | 1.6 | 5.9 | 0.1 | 0.790 | 4.76 | 2.9 | 5.0 | 0.3 | 0.557 | 18.88 |
| Mid-body | 5.8 | 5.1 | 1.3 | 0.250 | 329.28 | -1.0 | 4.0 | 0.1 | 0.795 | 0.35 |
| Posterior | -5.1 | 5.9 | 0.7 | 0.388 | 0.01 | 4.0 | 4.4 | 0.9 | 0.351 | 55.83 |
|  | Depression | |  |  |  | Depression | |  |  |  |
| Total | 3.0 | 7.0 | 0.2 | 0.665 | 20.41 | -1.1 | 5.5 | 0.0 | 0.845 | 0.34 |
| Anterior | 7.4 | 6.2 | 1.4 | 0.231 | 1685.36 | -1.5 | 5.3 | 0.1 | 0.775 | 0.22 |
| Mid-body | 4.7 | 5.3 | 0.8 | 0.376 | 111.60 | -5.6 | 4.2 | 1.8 | 0.181 | 0.00 |
| Posterior | -4.0 | 6.2 | 0.4 | 0.525 | 0.02 | -0.5 | 4.4 | 0.0 | 0.916 | 0.63 |
|  | Emotional Lability | |  |  |  | Emotional Lability | |  |  |  |
| Total | -2.2 | 8.3 | 0.1 | 0.793 | 0.11 | -6.2 | 6.5 | 0.9 | 0.347 | 0.00 |
| Anterior | -3.0 | 7.5 | 0.2 | 0.685 | 0.05 | -1.4 | 6.3 | 0.0 | 0.824 | 0.25 |
| Mid-body | -2.0 | 6.4 | 0.1 | 0.749 | 0.13 | -0.9 | 5.0 | 0.0 | 0.851 | 0.39 |
| Posterior | -4.5 | 7.5 | 0.4 | 0.547 | 0.01 | 0.6 | 5.3 | 0.0 | 0.908 | 1.84 |

Table S3. Equivalent to Table 5, but data set was limited to scans collected within one year of injury (n=402). Symptom presence relative to FA of corpus callosum regions. The logistic regression coefficient (estimate) and its standard error (SE) are shown. The chi-squared statistic and associated p-value and odds ratio (OR) are also provided.

| **Symptom** | **CC Region** | **Estimate** | **SE** | **X^2^** | **p** | **OR** |
| --- | --- | --- | --- | --- | --- | --- |
| **Headache** |  |  |  |  |  |  |
|  | Total | 3.8 | 8.2 | 0.2 | 0.645 | 43.96 |
|  | Anterior | 2.8 | 7.5 | 0.1 | 0.705 | 16.85 |
|  | Mid-body | -0.7 | 6.3 | 0.0 | 0.918 | 0.52 |
|  | Posterior | -8.9 | 7.4 | 1.5 | 0.221 | <0.01 |
| **Balance** |  |  |  |  |  |  |
|  | Total | -3.9 | 4.1 | 0.9 | 0.337 | 0.02 |
|  | Anterior | -5.2 | 3.6 | 2.0 | 0.154 | 0.01 |
|  | Mid-body | -3.4 | 3.1 | 1.2 | 0.280 | 0.03 |
|  | Posterior | -6.7 | 3.6 | 3.6 | 0.056 | <0.01 |
| **Cognitive** |  |  |  |  |  |  |
|  | Total | -3.5 | 4.2 | 0.7 | 0.411 | 0.03 |
|  | Anterior | -4.2 | 3.7 | 1.3 | 0.258 | 0.01 |
|  | Mid-body | -0.8 | 3.2 | 0.1 | 0.815 | 0.47 |
|  | Posterior | -3.4 | 3.6 | 0.9 | 0.344 | 0.03 |
| **Fatigue** |  |  |  |  |  |  |
|  | Total | -2.8 | 5.0 | 0.3 | 0.569 | 0.06 |
|  | Anterior | -5.3 | 4.6 | 1.4 | 0.244 | 0.01 |
|  | Mid-body | -1.7 | 3.8 | 0.2 | 0.658 | 0.19 |
|  | Posterior | -1.7 | 4.1 | 0.2 | 0.686 | 0.19 |
| **Anxiety** |  |  |  |  |  |  |
|  | Total | 0.4 | 4.3 | 0.0 | 0.934 | 1.43 |
|  | Anterior | 1.7 | 3.8 | 0.2 | 0.649 | 5.63 |
|  | Mid-body | 1.4 | 3.3 | 0.2 | 0.671 | 3.99 |
|  | Posterior | -0.2 | 3.6 | 0.0 | 0.948 | 0.79 |
| **Depression** | |  |  |  |  |  |
|  | Total | 0.2 | 4.5 | 0.0 | 0.966 | 1.21 |
|  | Anterior | 1.5 | 4.0 | 0.1 | 0.704 | 4.61 |
|  | Mid-body | -1.5 | 3.4 | 0.2 | 0.668 | 0.23 |
|  | Posterior | -1.7 | 3.8 | 0.2 | 0.652 | 0.18 |
| **Emotional** | |  |  |  |  |  |
| **Lability** | Total | -4.4 | 5.2 | 0.7 | 0.400 | 0.01 |
|  | Anterior | -2.3 | 4.8 | 0.2 | 0.635 | 0.11 |
|  | Mid-body | -0.9 | 4.0 | 0.1 | 0.814 | 0.39 |
|  | Posterior | -2.6 | 4.3 | 0.3 | 0.559 | 0.08 |

Table S4. Equivalent to Table 6, but data set was limited to scans collected within one year of injury (n=402). Symptom longevity relative to FA of corpus callosum regions. The permutation-based p-value and its 95% confidence interval (ci), and log rank score statistic are provided for each combination of region and symptom. The rows with p-value<0.05 are in bold.

| **Symptom** | **CC Region** | **p-value** | **p-value CI** | | **Score Statistic** |
| --- | --- | --- | --- | --- | --- |
| Headache (n=380) | |  |  |  |  |
|  | Total | 0.45 | 0.38 | 0.52 | 0.27 |
|  | Anterior | 0.39 | 0.33 | 0.46 | 0.35 |
|  | Mid-body | 0.21 | 0.16 | 0.26 | 0.57 |
|  | Posterior | 0.34 | 0.28 | 0.40 | 0.41 |
| Balance (n=278) | |  |  |  |  |
|  | Total | 0.98 | 0.89 | 1.00 | 0.00 |
|  | Anterior | 0.97 | 0.89 | 1.00 | 0.00 |
|  | Mid-body | 0.14 | 0.10 | 0.19 | 0.53 |
|  | Posterior | 0.20 | 0.15 | 0.25 | 0.43 |
| Cognitive (n=292) | |  |  |  |  |
|  | **Total** | **0.00** | **0.00** | **0.01** | **0.82** |
|  | **Anterior** | **0.00** | **0.00** | **0.01** | **0.91** |
|  | **Mid-body** | **0.02** | **0.01** | **0.04** | **0.94** |
|  | Posterior | 0.37 | 0.31 | 0.44 | 0.30 |
| Fatigue (n=69) |  |  |  |  |  |
|  | Total | 0.07 | 0.04 | 0.10 | 0.14 |
|  | Anterior | 0.41 | 0.34 | 0.47 | 0.07 |
|  | Mid-body | 0.93 | 0.85 | 1.00 | 0.01 |
|  | Posterior | 0.06 | 0.03 | 0.09 | 0.22 |
| Anxiety (n=106) | |  |  |  |  |
|  | Total | 0.07 | 0.05 | 0.11 | 0.22 |
|  | Anterior | 0.31 | 0.25 | 0.37 | 0.16 |
|  | Mid-body | 0.10 | 0.07 | 0.14 | 0.27 |
|  | Posterior | 0.23 | 0.18 | 0.28 | 0.18 |
| Depression (n=90) | |  |  |  |  |
|  | Total | 0.17 | 0.12 | 0.21 | 0.18 |
|  | Anterior | 0.10 | 0.07 | 0.14 | 0.23 |
|  | Mid-body | 0.34 | 0.28 | 0.40 | 0.17 |
|  | Posterior | 0.09 | 0.06 | 0.13 | 0.24 |
| Emotional Lability (n=60) | |  |  |  |  |
|  | Total | 0.06 | 0.03 | 0.09 | 0.15 |
|  | Anterior | 0.10 | 0.07 | 0.14 | 0.16 |
|  | Mid-body | 0.10 | 0.06 | 0.13 | 0.18 |
|  | **Posterior** | **0.00** | **0.00** | **0.01** | **0.29** |
